# Unanswered questions regarding the pathogenesis of late onset posterior capsular opacification

**DOI:** 10.3389/fopht.2025.1680042

**Published:** 2025-10-13

**Authors:** S. M. Rakib-Uz-Zaman, Liliana Werner, Melinda K. Duncan

**Affiliations:** 1 Department of Biological Sciences, University of Delaware, Newark, DE, United States; 2 John A. Moran Eye Center, University of Utah, Salt Lake City, UT, United States

**Keywords:** posterior capsular opacification (PCO), fibrotic PCO, pearl-like PCO, epithelial-to-mesenchymal transition (EMT), myofibroblasts, TGFβ, Soemmering’s ring, intraocular lens (IOL)

## Abstract

Following extracapsular cataract extraction, residual lens epithelial cells (LECs) are induced to express pro-inflammatory genes within hours of surgery, then begin to proliferate while migrating to populate denuded areas of the lens capsule. If these cells reach the optical axis, they scatter light, resulting in visual disturbances that are clinically defined as Posterior capsular opacification (PCO). Historically, PCO occurred at high rates within weeks or months of surgery, but over the past 10–20 years, this “acute onset” PCO has become relatively rare following cataract surgery in adults, due to improved surgical techniques and the ability of square edge intraocular lens (IOL) implants to block residual LECs from reaching the visual axis. Despite this, PCO rates are still substantial by 5–10 years following cataract surgery, apparently due to the ability of these entrapped cells to escape their confinement at the capsular bag periphery. This review explores the mechanisms by which cataract surgery elicits acute phenotypic changes to LECs and explores how these changes may set the stage for late-onset PCO.

## Cataract

Human vision requires the refractive properties of the transparent cornea and lens to collaborate in order to generate sharply defined images onto the retina, which then detects and processes these light signals prior to transmission to the brain (1–3). While opacification of either the cornea or lens compromises vision, defects in lens transparency, i.e., cataract, are historically the most common cause of human blindness (4). Cataracts can occur at any time across the lifespan and be triggered by any insult that disrupts lens anatomy, physiology, or biochemistry. However, cataract is predominately a disease of older adults as light scatter occurs when lens proteins and lipids are damaged by decades of exposure to reactive oxygen species and UV light, as well as spontaneous chemical reactions resulting in the formation of mixed disulfides and protein deamidation (5–9). In the earliest stages of age-related cataract, this light scatter leads to glare when driving at night, but later reduces light transmission to the retina, resulting in visual disability, and even complete blindness (9, 10).

## Cataract surgery history

As cataract-induced blindness greatly reduces quality of life, cataract treatment has long been a goal of medicine. The first effective cataract treatment, developed at the turn of the 20^th^ century, relied on intracapsular cataract extraction (ICCE), removal of the entire lens through a large 10-12mm incision (11, 12). This procedure required a long recovery period with minimal activity, while visual restoration required the patient to wear heavy, high diopter spectacles to compensate for the loss of the lens’s refractive power from the aphakic eye (12). Beyond these functional limitations, ICCE presented serious ocular risks that restricted its use to advanced cataracts. A complete aphakic state allows the aqueous and vitreous humors to mix, increasing the risk of vitreous prolapse and subsequent retinal detachment; studies show aphakic eyes have a 3-5% lifetime risk of retinal detachment compared to 0.01-0.1% in phakic eyes (13–16). Additionally, the large corneal incision frequently induced significant astigmatism (typically 4–6 diopters) and carried risks of wound dehiscence (12, 17). These complications, combined with the frequent development of cystoid macular edema (occurring in 15-30% of cases), meant the procedure was typically reserved for patients with mature cataracts who had already endured years of visual disability (12, 17).

The first attempts to restore the eye’s refractive power following cataract extraction emerged in the mid-20^th^ century. British Royal Air Force pilots who had acrylic plastic fragments from shattered cockpit canopies embedded in their eyes during World War II showed remarkable tolerance to the foreign material (11, 12, 18). This serendipitous discovery led to the development of the first intraocular lens (IOL) made of polymethyl methacrylate (PMMA), which was implanted several months following extracapsular cataract extraction (ECCE) that removed the central anterior capsule and fiber cell mass, while preserving both the equatorial anterior and posterior lens capsule (capsular bag) (11, 18). These early IOLs, while revolutionary, presented significant challenges. The original Ridley lens was placed in the posterior chamber, but the procedure’s technical difficulty and frequent complications led to modifications (12). Anterior chamber IOLs were developed for placement in the anterior chamber or with iris fixation to correct residual refractive error, but the ocular damage induced by their placement often caused uveitis-glaucoma-hyphemia (UGH) syndrome, corneal endothelial damage, and persistent inflammation (19–25). In the 1970s, ECCE was re-introduced with the IOL placed within the capsular bag at the time of initial surgery so that the capsule’s attachment to the ciliary muscle via the zonules fixed its location within the eye while minimizing damage to other ocular structures (12, 26, 27).

## Posterior capsular opacification after ECCE

**Figure 1:**
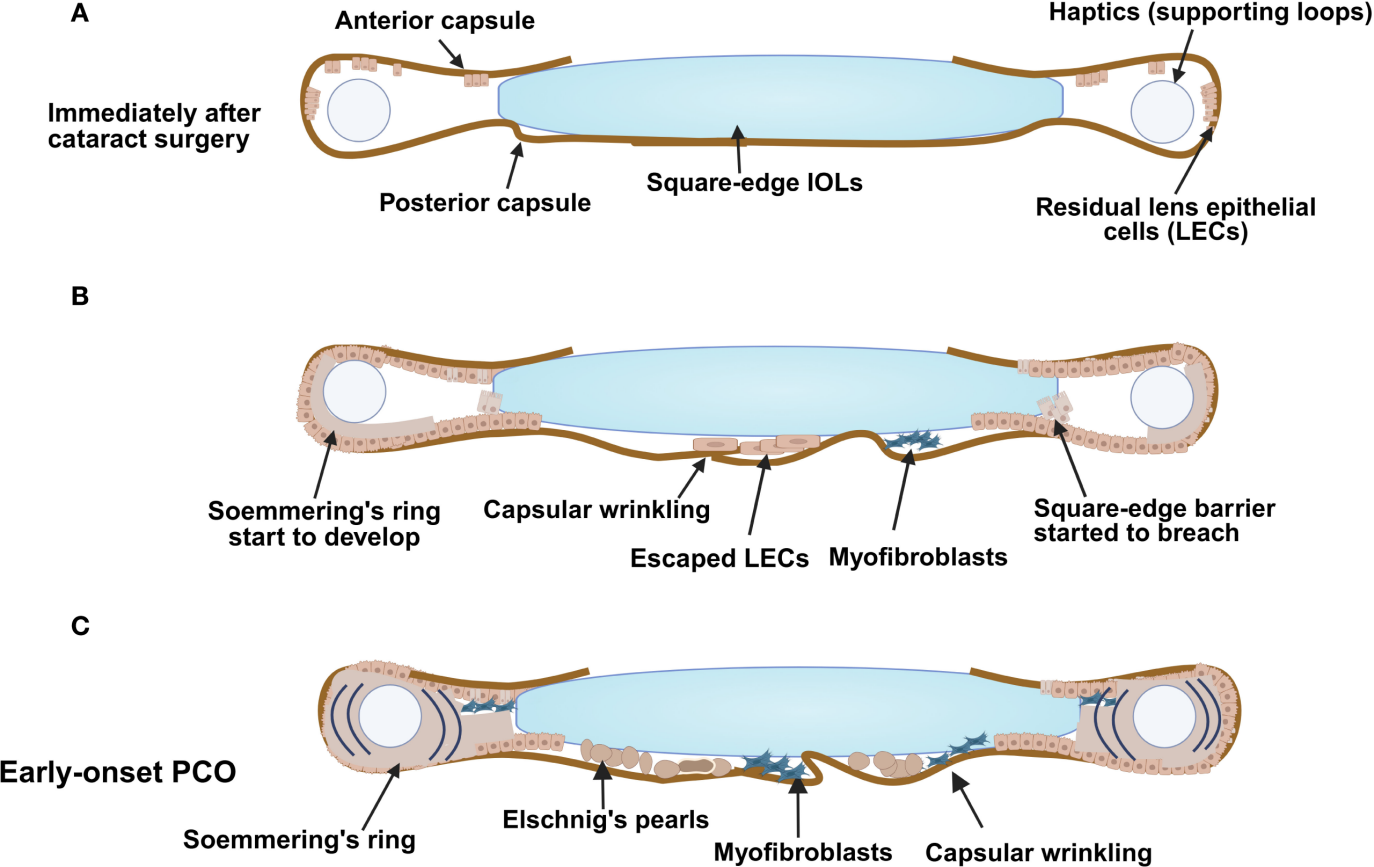
Early-onset PCO development following cataract surgery. An illustration demonstrating **(A)** the capsular bag early after surgery, **(B)** cells start to gather at the posterior region (some pearl-like components and some myofibroblasts) and development of matrix deformation/capsular wrinkling; Soemmering’s ring also starts to appear and **(C)** the changes that result from a combination of fibrotic and regenerative PCO with formation of Elschnig’ s pearls and Soemmering’s ring. *Figure created with Biorender*.

While ECCE with IOL implantation improved cataract surgery outcomes, lens epithelial cells (LECs) (28) retained in the capsular bag following cataract surgery due to their attachment to the equatorial lens capsule, undergo a wound healing response that induces them to proliferate and migrate onto the denuded capsule while differentiating into a mixed population of disorganized lens fiber cells and myofibroblasts (29–31). If these cells remain in the periphery of the capsular bag, they form Soemmering’s ring, which increases the long-term stability of the IOL within the eye (31–34). However, if these cells migrate into the optical axis (ie, central posterior capsule), they interfere with vision both due to their intrinsic tendency to scatter light and ability to wrinkle the posterior capsule (**Figure 1**) (34–38). Initially, PCO was a major impediment to the routine clinical use of ECCE as clinically significant visual disturbances due to PCO would routinely develop within weeks or months of cataract surgery which could only be treated by invasive posterior capsule removal surgery (12, 32, 39–41). Historical reports from the 1980s and early 1990s indicate that the incidence of clinically significant PCO following ECCE reached as high as 30–50% after 1 year PCS (42, 43). In the early 1980s, the clinical impact of PCO was greatly reduced by the development of Nd: YAG laser capsulotomy, which can non-invasively ablate the posterior lens capsule and attached light-scattering cells to restore transparency of the optical axis (32). However, YAG laser capsulotomy is not entirely benign as it releases cellular material into the posterior chamber which can trigger inflammation leading to cystoid macular edema/retinal detachment, as well as exacerbation of pre-existing uveitis or glaucoma (32, 44–47). Thus, it was also desirable to reduce overall PCO rates to further improve the outcomes of cataract surgery.

## Cataract surgery in 2025

Modern cataract surgery has evolved significantly to minimize the incidence of PCO, with numerous innovations which have primarily focused on reducing both postoperative inflammation and the potential of residual lens-derived cells to reach the visual axis (12, 48, 49). Currently, small-incision phacoemulsification cataract removal is standard in clinical care as it minimizes the ocular trauma that appears to trigger some of the acute post-operative inflammation which drives the most severe potential acute complications of cataract surgery including cystoid macular edema, retinal detachment, and chronic inflammation/uveitis while also reducing the rate of clinically significant PCO in the first year post surgery (7, 12, 47, 48, 50–53).

**Figure 2:**
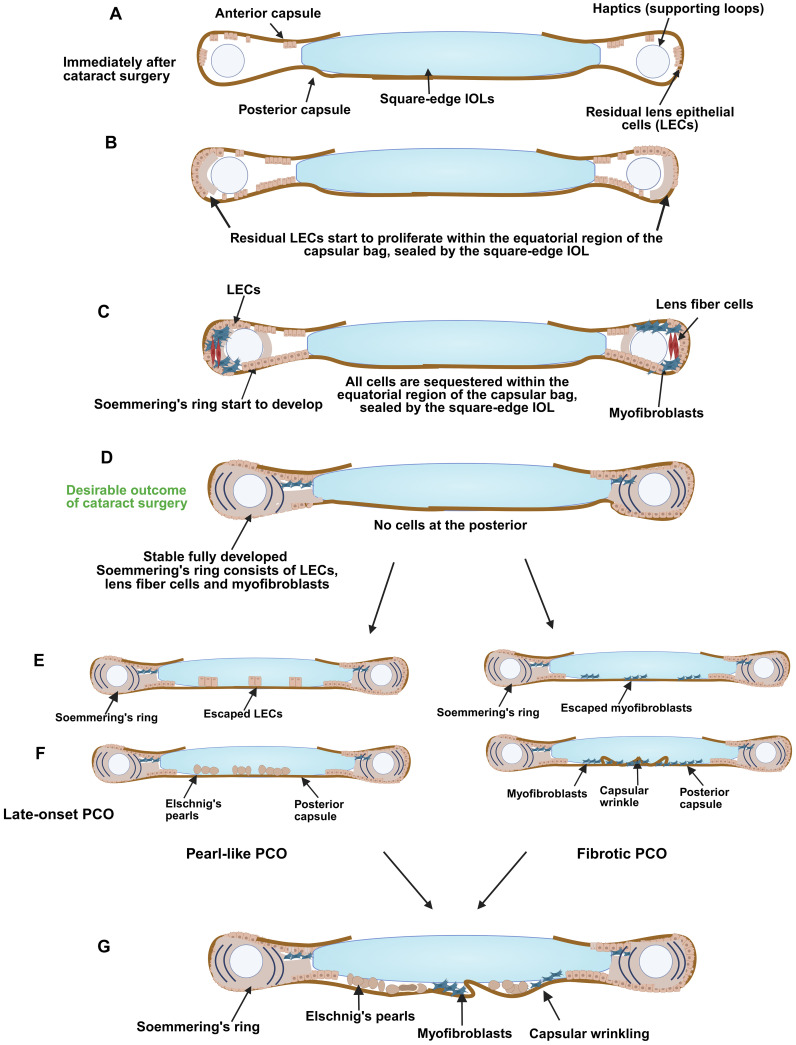
Development of late-onset PCO. A schematic diagram illustrating **(A)** the capsular bag early after surgery, **(B)** LECs start to proliferate and populate the areas of denuded peripheral lens capsule that they can access, **(C)** Soemmering’s ring starts to develop, **(D)** Soemmering’s ring fully develops, and cells start to gather at the posterior region (myofibroblasts or LECs), **(E)** development of late-onset PCO showing differentiation of escaped LECs into Elschnig's pearls (left) or invasion of lecs or myofibroblasts onto the inner surface of the posterior capsule the posterior capsule, differentiation of escaped LECs into Elschnig's pearls (left) or and matrix deformation/capsular wrinkling due to population of the posterior capsule with myofibroblasts (right), **(F)** Late-onset PCO is formed: it can be pearl-like (left) and/or fibrotic (right) in nature, or be a mixture of these types **(G)**. *Figure created with Biorender*.

In this procedure, a 2.2 mm or smaller incision is made in the peripheral corneal to access the cloudy lens. This small opening causes less disruption to the eye’s structure, reduces surgical trauma, and allows for faster healing. The use of a small incision also helps prevent postoperative astigmatism (corneal distortion) and lowers infection risks (12). After creating the incision, a viscoelastic agent (usually a high molecular weight hyaluronic acid solution) is injected to stabilize the anterior chamber while protecting the cornea endothelium from biomechanical damage. The central lens capsule with attached lens epithelial cells is then separated from the lens (anterior capsulotomy), either by manually tearing using forceps (continuous curvilinear capsulorhexis (CCC)), or by use of a femtosecond laser, which creates the access needed for lens fiber cell removal and IOL implantation (54). The typical anterior capsulotomy used in adult cataract surgery ranges from 5-5.5 mm in diameter, as this is slightly smaller than the optic component of the IOL. Clinical studies show that appropriate sizing of the anterior capsulotomy reduces PCO rates, apparently due to enhanced adhesion between the lens capsule and IOL, which may reduce the access of residual LECs to pro-inflammatory cytokines and growth factors present in the aqueous humor (55, 56). Additionally, this technique induces the lens capsule to “shrink-wrap” around the IOL which helps fix the IOL into the correct location within the eye which also likely inhibits LEC proliferation via contact inhibition while creating a barrier to LEC migration onto the central posterior capsule (**Figure 2D**) (57, 58).

After anterior capsulotomy, the fiber mass is separated from the capsule by injection of balanced saline solution (BSS), a procedure known as hydrodissection; then the cortical fibers are separated from the central lens fiber mass (lens nucleus) by forcing BSS into the fiber mass (hydrodelineation). The nuclear fiber mass can be further broken down by various chopping techniques, and the cellular material is then pulverized into a cellular slurry via ultrasonic vibration (phacoemulsification) which is removed by simultaneous suction aspiration. Any residual cortical fibers left in the capsular bag after dealing with the nuclear material are removed via irrigation/aspiration. Clinical studies have found that both minimizing biomechanical forces on the eye during phacoemulsification and maximizing the removal of peripheral lens material (cortical cleanup) reduce short term PCO rates, perhaps by reducing the stimuli that can drive persistent tissue inflammation following surgery (see below) (59–63). Once all apparent cellular material is removed from the capsular bag, an IOL is inserted to restore refractive function. The viscoelastic is then removed from the eye, and the small incision(s) allowed to self-seal without the placement of sutures. The short-term visual outcome of this procedure is excellent, with studies reporting that approximately 87% to 98% of patients achieve non-spectacle (uncorrected) visual acuity of 20/60 (equivalent to 6/18 in metric units) or better within six weeks post-surgery (64, 65).

## Modern IOL designs also reduce acute PCO incidence

Permanent placement of artificial implants into the human body is generally challenging due to their tendency to trigger the “foreign body” response, which is a chronic inflammatory reaction to the material of the implant (66–68). IOLs were one of the first successful long-term medical implants due to the immune system’s general tolerance for the plastics used (11, 68–70). However, numerous studies have found that PCO rates are still heavily influenced by the material properties of the plastic that makes up the IOL, with the lowest PCO rates occurring when the IOL adheres tightly to the posterior lens capsule, likely creating a physical barrier that prevents the migration of cells onto the portion of the posterior capsule within the visual axis, commonly known as the “no space, no cells” theory of PCO prevention (49, 71, 72).

IOLs made from hydrophobic acrylic significantly reduce the migration of LECs onto the posterior capsule compared to older materials like polymethyl methacrylate (PMMA) or silicone (69, 72–74) due to their ability to preferentially adsorb ECM proteins, notably fibronectin, from the surrounding ocular environment, which then crosslink with collagen IV in the lens capsule (75, 76). The acrylate groups in hydrophobic IOLs also form covalent bonds with the cysteine-rich domains common in capsular proteins (77). This early adhesion encourages the formation of a capsular bend, a sharp inward fold where the edge of the capsule wraps around the IOL (57, 78). This fold, combined with tight adhesion, acts as a physical barrier that blocks LECs from moving into the visual axis (57, 78). In contrast, IOLs made from hydrophilic acrylic tend to form a weaker bond with the capsule (water-rich boundary layer in hydrophilic acrylic IOLs sterically hinders fibronectin absorption), resulting in poorer mechanical integration (68, 69, 73, 79, 80). This weaker adhesion leaves a microscopic gap between the IOL and the capsule surface, providing an open route for LECs to migrate and proliferate, leading to higher PCO rates (79, 80).

**Figure 3:**
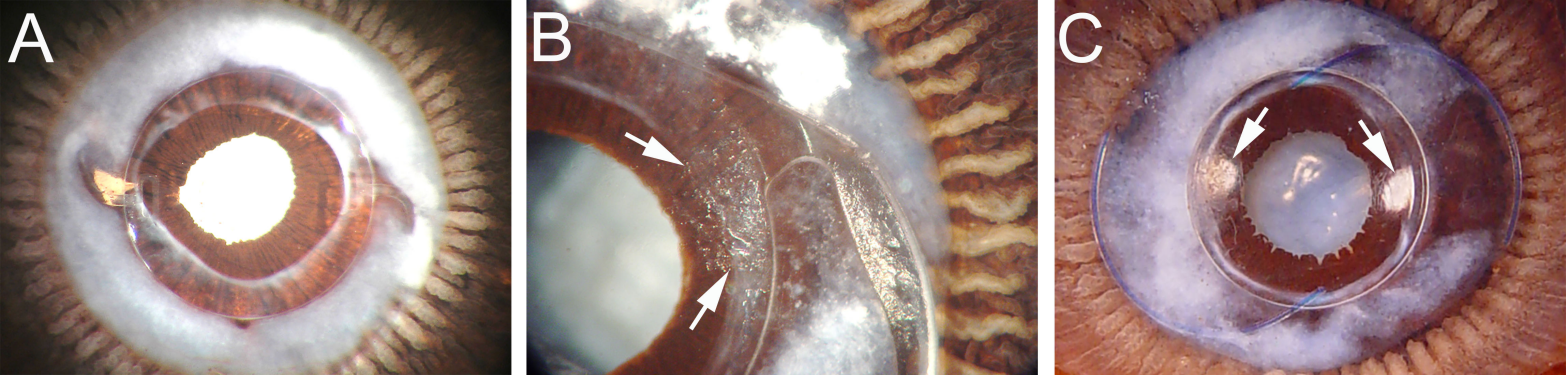
Miyake-Apple views of the posterior capsule of cadaver lenses at extended times post cataract surgery **(A)** Eye implanted with a 3-piece silicone square edge IOL 7.58 years prior to death. This eye exhibits a prominent Soemmering’s ring with fibrosis apparent at the rim of the capsulorhexis opening, but no PCO. **(B)** Eye implanted with a 1-piece square edge hydrophobic acrylic IOL at 2.25 years prior to death. Peripheral PCO has begun that emanates from the optic-haptic junction (Arrows). **(C)** Eye implanted with a 3-piece square edge hydrophobic acrylic IOL at 2.33 years prior to death. This eye exhibits a less dense Soemmering’s ring, but peripheral PCO is seen at two sites at approximately 3 and 9 o’clock (Arrows). Details of sample preparation can be found in (84).

While the chemical properties of the materials from which IOLs are made influences acute PCO rates, the “no space, no cells” principle also drives IOL design as PCO can only occur if residual lens cells can proliferate to numbers able to scatter light while migrating from their native location on the peripheral anterior lens capsule to the central posterior capsule (39, 71, 72). Currently, most IOLs are designed to include optics with “square” outer edges that are capable of sealing tightly to the lens capsule so that residual lens cells encounter a physical barrier to their migration onto the posterior capsule (55, 81). The cells thus entrapped at the lens periphery also exhibit reduced cell proliferation, likely due to “contact inhibition” which is a critical mechanism that epithelial cells use to prevent cellular overgrowth (82, 83). However, over time, cell growth does still occur, leading to Soemmering’s ring, an opaque ring of cells deposited around the IOL (See **Figure 3A**) (31, 33, 85). Soemmering’s ring does not typically interfere with vision as it is located outside of the visual axis (33). It may even be beneficial for long term fixation of IOL position, as well as long-term maintenance of the lens capsule, as cataract patients who lack Soemmering’s ring (a condition called “Dead bag syndrome”) often experience late-stage cataract surgery side effects including late lens dislocations (see below) (33, 86, 87).

While the “no-space, no-cells” concept emphasizes that intimate capsule–IOL apposition limits LEC ingress and PCO (88), observations from the human capsular-bag model indicate that true cell–IOL engagement is not uniform across the optic (89). In a graded-culture human capsular-bag system, LECs tend to adhere and/or grow to some degree on the anterior IOL face within the capsulorhexis margin but show limited engagement with the remaining IOL surface (89).

It is likely that both IOL material and IOL design work together to reduce PCO incidence associated with IOLs made from hydrophobic materials, as it was initially difficult to create hydrophilic IOLs with consistent sharp edge architecture, although square edge silicone lenses are now available and also exhibit reduced PCO rates (90). There is also emerging evidence that optimizing the geometry of the posterior side of the optic to match the curvature of the normal human lens can also reduce PCO rates, likely due to increased IOL-capsule adherence (71, 91, 92). Despite these advances, PCO still occurs at appreciable rates following cataract surgery. In some cases, this is due to uneven sealing of the capsule to the IOL, perhaps due to an uneven capsulorhexis. In others, it appears that single piece IOL designs that integrate the haptic (which fixes the IOL into the capsular bag) with the optic do not create a robust seal between the optic and the lens capsule (**Figure 3B**) (84, 93, 94). However, PCO still occurs in some patients whose surgeries generated ideal capsule geometry and were implanted with hydrophobic square edge three-piece IOLs where the square edge barrier is uninterrupted (**Figure 3C**) (84, 95).

## Subclinical versus clinically relevant PCO

PCO is defined as the presence of light-scattering elements on the portion of the posterior capsule found within the visual axis (31, 96). However, it may not significantly affect the patient’s quality of life, particularly if it remains at the periphery of the visual axis and/or is at low density (97, 98). In most contexts, clinically relevant PCO is pragmatically defined as that which creates light scatter severe enough to affect a patient’s life activities (such as onset of glare that interferes with night driving or reduced overall visual acuity) (99–101) (102). However, even severe PCO may not be considered clinically relevant if it is present in a patient whose vision is already impaired for other reasons such as age-related macular degeneration or glaucoma (103, 104). In research settings, PCO can be quantified using validated imaging methods such as POCOman (105) or AQUAII (106) to assess its severity and visual impact. This allows “clinically significant (vision-threatening) PCO”, which prompts treatment with Nd: YAG laser capsulotomy, to be distinguished from “subclinical PCO” where light scattering within the visual axis is detected but does not significantly impact the patient’s subjective vision (49, 107).

It should be noted that most large clinical and registry studies of PCO rates use Nd: YAG capsulotomy as a proxy for the presence of clinically significant PCO, and these data also underly the predictive models that estimate risk of a patient undergoing cataract surgery will require Nd: YAG over time (102, 107). As different health care systems use different thresholds for “clinically significant PCO”, including whether patients experiencing severe cognitive decline or other major health issues are candidates for treatment, this can lead to differences in reported PCO rates among populations to arise solely from differences in clinical decision making (108, 109). Further, subclinical PCO is also relatively difficult to assess in assess in community health care settings and the resulting underreporting in medical records means that the rate that “subclinical” PCO progresses to later “clinically significant” PCO is generally understudied.

Thus, in this review, reported PCO “rates” are generally referring to PCO that was severe enough to be treated by Nd: YAG laser capsulotomy or other methods. In contrast, the sections focused on the cataract surgery-induced changes in cell biology that lead to PCO are relevant to both subclinical and clinically relevant PCO as well as the production of cells sequestered in Soemmering’s ring or other locations outside of the visual axis such as those associated with the equatorial anterior capsule.

## Current reported rates of early onset PCO

Early-onset PCO, also referred to as acute PCO, is defined as opacification that develops within the first year following cataract surgery. Its incidence has substantially reduced over the past decade, largely due to advancements in surgical techniques, IOL design, and postoperative care (39, 42, 49, 110). Large population-based studies now report clinically significant early-onset PCO in approximately 2–4.5% of adult patients within the first postoperative year, a marked improvement compared to historical rates of 10–20% prior to 2000 (**Table 1**) (107, 119). The most pronounced reductions have been observed in patients receiving hydrophobic acrylic IOLs with 360° square-edged optics (**Table 2**), particularly when combined with complete cortical cleanup and an optimized capsulorhexis–optic overlap (30, 110, 124, 125). Prospective studies utilizing these methods have reported 1-year PCO rates as low as 1.1% (**Table 2**) (120). In contrast, higher rates of early-onset PCO persist in patients implanted with silicone IOLs, with incidences ranging from 11% to 40.7% reported as early as 6 weeks to 3 months postoperatively (**Table 2**) (107, 110, 126, 127), although advances in silicone IOL manufacturing have now led to the release of square-edged silicone IOLs with lower rates (90, 128).

**Table 1:** Early-onset PCO rates (≤1 Year following cataract surgery) varies by age.

Age group	1-year PCO rate	Key risk factors	References
Infants (<1 yr)	58% to 100%	Congenital cataracts, no primary capsulotomy	(32, 48, 49, 111, 112)
Children (1–17 yrs)	22-70%	Trauma, silicone IOLs	(113, 114)
Young/Adults (18-59)	11.8-28%	High myopia (>6D), Type 1 diabetes	(115–118)
Elderly (>60 yrs)	4.4-29.9%	Pseudoexfoliation, small pupil, high myopia	(107, 110, 115)

**Table 2:** Incidence of early-onset PCO depending on IOL material.

IOL type	PCO rate	References
Hydrophobic acrylic	1.1-3.6%	(107, 120, 121)
Hydrophilic acrylic	11.9%	(107, 121)
Silicone	11-40.7%	(110)
PMMA	11.8-30%	(119, 122, 123)

Certain patient populations demonstrate disproportionately high susceptibility to early-onset PCO. Pediatric patients exhibit the highest incidence, with reported rates ranging from 40% to 100% at one-year post-surgery (**Table 1**) (32, 48, 49, 111, 112). This elevated risk is driven, at least in part, by biological factors such as the heightened proliferative capacity of pediatric LECs, increased TGFβ and fibroblast growth factor (FGF) signaling, and more robust postoperative inflammatory responses (32, 117, 129, 130). However, a mismatch between the posterior curvature of the pediatric lens and the adult designed IOLs used “off label” in these patients likely also influences this enhanced PCO rate as this would compromise the seal between the IOL and posterior lens capsule consistent with the “no space, no cells” principle for PCO prevention (88). Young adults show intermediate risk, with PCO rates ranging from 11.8% to 28%, again likely due to a more robust wound healing response (**Table 1**) (115–118).

Ocular co-morbidities can also lead to a higher incidence of PCO following cataract surgery. Individuals with high myopia exhibit higher PCO rates likely due to biological factors like higher levels of intrinsic ocular inflammation (107, 131). However, these patients also experience a geometric mismatch between the IOL and posterior lens capsule as they are implanted with lower-power IOLs with less posterior curvature to achieve spectacle-free 20:20 vision (92, 132). This is likely exacerbated by the weakened zonular fibers common in high myopia, which would lead to poor capsular tension and further reduced IOL-capsule adhesion (131, 133). In uveitis, chronic ocular inflammation elevates cytokine (IL-6, TGFβ) and matrix metalloproteinase (MMP-2 and MMP-9) levels, which collectively degrade the capsular basement membrane, stimulate LEC proliferation, and promote EMT (134–138). The known cellular responses to cataract surgery that likely contribute to PCO are described below.

## PCO is still prevalent in elderly patients at prolonged times following cataract surgery

While advanced IOL designs that sequester remnant LECs outside of the visual axis and surgical techniques that minimize ocular trauma/inflammation have significantly reduced the rate of clinically significant PCO development over the first year PCS for those treated for age-related cataract, substantial numbers of these patients still develop PCO at longer times PCS (107, 121, 139, 140) (**Table 3**). Further, similar to acute PCO, late-onset PCO is more common in patients with ocular co-morbidities such as high myopia (axial length ≥26 mm), Marfan’s syndrome, and Chronic Uveitis (**Table 4**) (145, 147, 149). Late-onset PCO is also more common in patients implanted with hydrophilic IOLs (107, 121, 142). Despite these similarities between acute and late-onset PCO, it should be noted that late-onset PCO typically occurs in “quiet” eyes that completed “active” wound healing in response to cataract surgery years prior (150). Below, we explore what is currently known about the acute response of residual LECs to cataract surgery and present hypotheses about how this initial response can set the stage for late-onset PCO.

**Table 3:** Incidence of long-term PCO in eyes implanted with IOLs of different materials.

IOL materials	5 years (%)	9 years (%)	References
Hydrophobic	25.2	39.3	(107, 141, 142)
Hydrophilic	51.4	63.1	(107, 141, 142)
Silicone	19.4	30.8	(107, 142)
Mixed (Hydrophobic+ Hydrophilic)	67.7	N/A	(107)
PMMA	56	N/A	(107, 143)

**Table 4:** Late-onset PCO in eyes with ocular co-morbidities.

Condition	PCO rate (%)	Time frame	References
High Myopia (Axial > 26 mm)	14.8% – 56.8%	2–4 years	(144–146)
Marfan’s Syndrome	23-69%	Up to 5 years	(147, 148)
Uveitis	>55%	Within 3 years	(149)
Cystoid macular edema	~16%	Within 3 years	(149)

## Current understanding of the molecular basis of short-term/acute PCO

Small incision cataract surgery removes the central lens capsule and attached LECs, then uses phacoemulsification to remove the fiber cell mass (7, 31, 151). However, the equatorial LECs are resistant to removal due to both their tight adherence to the lens capsule and location relative to the surgical incision (**Figure 1**) (49, 117). It is accepted that PCO material is largely derived from these residual LECs, and it has long been understood that enhanced growth factor signaling (TGFβ, FGF and others) induced by cataract surgery drives their proliferation, migration, and differentiation into either aberrant fiber cells (cellular basis of Elschnig’s “pearl-like” PCO) or myofibroblasts (cell type responsible for fibrotic PCO) (31, 32, 152–156). However, the mechanisms by which cataract surgery induces these processes have been traditionally less investigated.

**Figure 4:**
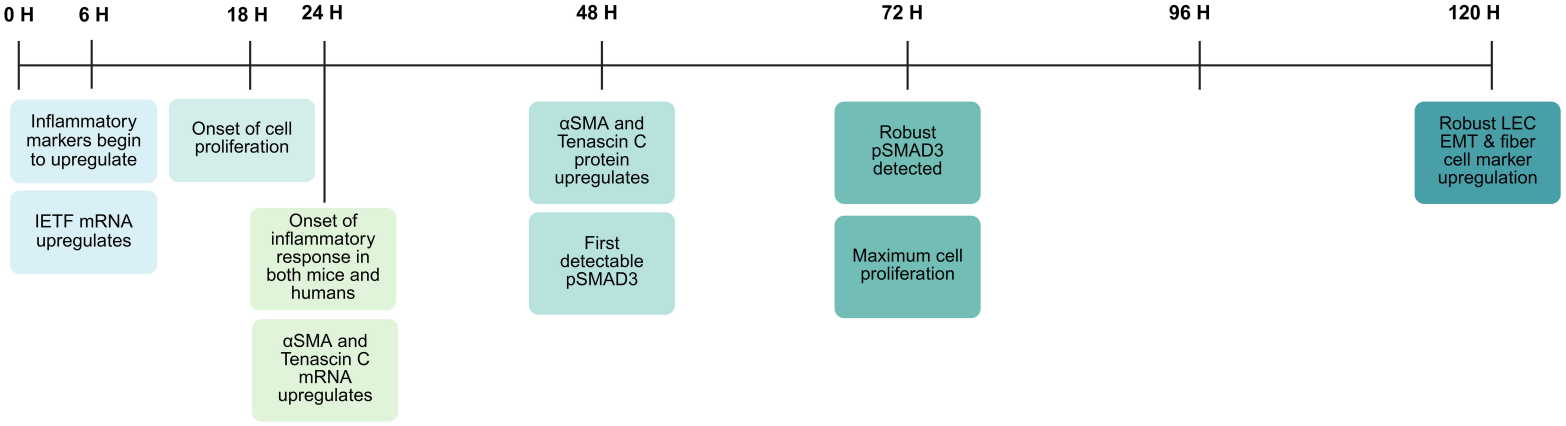
Injury response time course in lens epithelial cells PCS in an *in vivo* mouse model of cataract surgery (184). *Figure created with Biorender*.

LEC cultures are used to investigate the cell signaling pathways responsible for the phenotypic conversion of LECs to the myofibroblasts and fiber-like cells that comprise PCO material (117, 157–161). While these approaches have garnered important information about the pathways regulating some of the cellular responses leading to PCO, established lens epithelial cell lines have a profoundly different transcriptome than bona fide LECs residing within the mammalian lens, and often have dysregulated cell cycles resulting from either transformation with SV40 or just selection for growth in culture (162–166). Many of these limitations can be overcome by the use of primary LEC cultures established by lens dissociation (167, 168), LEC explants grown on their native lens capsule (169–174), or the human lens capsular bag model where cadaver lenses are subjected to similar manipulations as occur during cataract surgery, followed by culture (58, 175–178). However, they are only able to model the response of LECs to the *in vitro* cell culture environment without consideration of the complexity of the ocular or wider organismal response to the surgery, so these investigations are enhanced by the use of animal-based *in vivo* cataract surgery models including monkeys (179), pigs (180), rabbits (181), rats (182) and mice (183–185). Here we outline what is currently known about timing (**Figure 4**) and nature of the molecular events occurring in LECs following cataract surgery that are likely to contribute to acute onset PCO.

### LECs rapidly upregulate the expression of pro-inflammatory cytokines in response to lens manipulations/injury or cataract surgery

Cataract surgery induces a phenotypic change in the retained peripheral LECs, which leads to their proliferation, migration, and differentiation into the myofibroblasts and dysgenic lens fibers that contribute to Soemmering’s ring, and PCO (30, 89, 186). However, it was believed that LECs are not primarily affected by surgical manipulations or placement into explant culture; instead, the phenotypic changes seen in these cells days after surgery derive from the LEC response to the environment of the eye (173, 187, 188). This view was supported by numerous studies that found rat LEC explants (173, 189, 190) or human LECs (178, 191) cultured in the capsular bag model largely retain their epithelial character in the first few days of culture, but respond robustly to treatment with numerous growth factors, most notably active TGFβ (189, 190) or heparan bound FGF (190–192). However, an early report suggested that LECs rapidly change crystallin localization upon lens isolation (193), and now RNAseq experiments have shown that chicken (194), mouse (61, 195), rat (196), and human (160, 197, 198) LECs remodel between 10-33% of their transcriptome within a day of isolation including a robust upregulation of immediate early transcription factors and proinflammatory cytokine expression upon either isolation in culture (194), or following lens fiber cell removal surgery *in vivo* (61, 195). By 24 hours post lens injury, LECs are producing high levels of numerous proinflammatory cytokines proteins including CXCL1, S100a9, CSF3, COX-2, CCL2, LCN2, and HMOX1 which correlates temporally with neutrophils reaching the eye between 18–24 hours post lens injury in a mouse *in vivo* model of cataract surgery (**Figure 4**) (160, 199), and the onset of maximal “flare” a measure of ocular inflammation, in humans following cataract surgery (200–202).

Historically, it was believed that ocular inflammation post cataract surgery arose exclusively from mechanical disruption of the blood-aqueous barrier (BAB) during surgery, which allows serum proteins and immune cells to enter the eye (166, 203). However, this postulate does not consider how expression of pro-inflammatory cytokines by other ocular structures, including residual LECs, could initiate loosening of the BAB tight junctions, as is seen in other injured tissues such as the gut (204) and heart (205), to create aqueous flare following cataract surgery (61). The relative contributions of direct mechanical distortion of the tissues comprising the BAB and loosening of BAB tight junctions in response to the release of pro-inflammatory cytokines by injured ocular tissues on the ocular inflammatory response following cataract surgery require future investigation.

While the molecular mechanisms by which experimental or surgical manipulation of the lens drives the rapid upregulation of pro-inflammatory cytokine expression in LECs are currently unclear, LECs do rapidly induce Erk phosphorylation when isolated from animals (61) and this pathway is known to directly trigger immediate early transcription factor expression in other tissues (206, 207). Future studies are needed to determine how/why lens manipulations trigger Erk activation, and how this pathway influences later injury responses.

### The onset of the LEC fibrotic response after lens injury

The transcriptional changes induced in LECs within 6 hours of lens injury in mice subjected to lens fiber cell removal surgery do not include fibrotic markers associated with LEC conversion to myofibroblasts (61). However, these genes are robustly upregulated at the mRNA level within 24 hours PCS in both rodent (196) and human culture models (160) as well as following *in vivo* lens injury in mice (183, 199). This appears to be a conserved response/timeline in mammals as a cross-species comparison of transcriptomic changes in mouse and human LECs at 24 hours post fiber cell removal revealed that 23 genes from the “HALLMARK EMT” gene set were commonly regulated, including classical EMT markers such as αSMA and tenascin C (160). Notably though, neither morphological remodeling of LECs into myofibroblasts nor the upregulation of fibrotic marker protein levels are detected at 24 hours post lens injury *in vivo* (183). However, by 48 hours PLI in the *in vivo* mouse lens fiber cell removal surgery model, variable amounts of enhanced fibrotic protein expression are associated with LECs including polymerized αSMA, and fibrotic ECM molecules such as fibronectin and tenascin C (183, 185, 199). By 72 hours PCS, fibrotic protein upregulation is established, lens epithelial marker expression has downregulated, and remnant lens cells undergo a burst of cell proliferation (**Figure 4**) (199, 208). At 5 days PCS and beyond, there is the sustained survival of cells with a myofibroblast phenotype as measured by morphology, high levels of αSMA fibrils, robust production of fibrotic ECM, along with low levels of typical LEC markers such as E-cadherin and Pax6 (**Figure 4**) (183, 199, 208, 209).

### Known molecular mechanisms driving LEC fibrosis following lens injury/cataract surgery

Currently, it is generally accepted that production of fibrotic tissue following lens injury leading to anterior subcapsular cataract, or after cataract surgery to create cells capable of contributing to fibrotic PCO, is driven by transdifferentiation of LECs to myofibroblasts via epithelial to mesenchymal transition (36, 49, 153, 185). However, it has been proposed that at least a portion of the myofibroblasts found associated with capsular bags post cataract surgery are derived from a tissue resident population of epiblast-derived MyoD/Nog cells, as capsular bag fibrosis is reduced upon depletion of this cell population (210, 211). It is also plausible that some of the myofibroblasts detected post lens injury are derived from differentiation of circulating fibrocytes, which reach the lens during the acute inflammatory response to injury (212, 213). While definitive answer to the source of all myofibroblasts that are associated with the lens following its injury will likely await genetic lineage tracing approaches, much is known about the cell signaling pathways required for myofibroblast production and prolonged maintenance following injury/surgery, all of which are potential entry points for the development of drug based therapeutic interventions capable of reducing PCO incidence following cataract surgery (185, 214–217).

### TGFβ-mediated processes drive myofibroblast production after lens injury/surgery

It is widely recognized that TGFβ is a major player driving the pathogenesis of fibrotic conditions throughout the body, including both anterior subcapsular cataract and PCO (153, 167, 215). Mammalian TGFβs are a family of three related proteins (TGFβ1, TGFβ2, and TGFβ3) that are produced by cells in a latent form (218, 219). Latent TGFβ originates in the endoplasmic reticulum, where pro-TGFβ and its associated latency-binding protein (LTBP) are co-translated (220). Pro-TGFβ undergoes dimerization and is covalently linked to LTBP through disulfide bonds, forming the large latent complex (LLC) (220, 221). TGFβ is cleaved from its pro-domain, the LAP, in the trans-Golgi network, where they remain noncovalently bound and continue to form the LLC (221). After secretion, the LLC can be found in both body fluids or bound to extracellular matrix components, where it can remain inactive for substantial lengths of time (221).

In order to trigger signaling, latent TGFβ requires “activation” which involves separation of the functional growth factor from the LTBP and LAP (183, 220), a process that can occur via diverse mechanisms. The released active TGFβ then binds to the type II TGFβ receptor to form an active receptor complex (183). In canonical TGFβ signaling, the receptor complex phosphorylates the type I receptor, which activates the SMAD anchor receptor activation (SARA) to recruit SMAD 2 or SMAD 3 to the type I receptor (183). Once recruited, SMAD2/3 are phosphorylated and form heteromeric complexes with the co-mediator SMAD4 (183, 222). These complexes then translocate into the nucleus, where they drive the transcription of pro-fibrotic genes while suppressing epithelial gene expression, thereby initiating EMT and subsequent fibrosis (183). In addition to this canonical SMAD-mediated pathway, non-canonical TGFβ-induced signaling pathways, notably the MAPK/ERK, appear to also play critical, synergistic roles in the induction and progression of EMT in LECs (167, 223). TGFβ can activate the Ras–Raf–MEK–ERK pathway via tyrosine phosphorylation of the adaptor protein Shc upon binding to TGFβRI (224). This phosphorylation event enables formation of the ShcA/Grb2/SOS complex, leading to Ras activation, sequential kinase signaling, and ERK1/2 phosphorylation, which contributes to EMT-associated transcriptional events (224). Simultaneously, TGFβ induces activation of the p38 and JNK MAPKs through a distinct SMAD-independent mechanism involving TRAF6 and TAK1 (MAP3K7) (225). Upon TGFβ receptor engagement, TRAF6 undergoes K63-linked polyubiquitylation and associates with TAK1, facilitating downstream phosphorylation of MKK4/6 and subsequent activation of JNK and p38 (226). These kinases then phosphorylate SMAD linker regions, amplifying fibrotic and EMT responses independently of canonical SMAD activation (225, 226).

Notably, phosphorylated SMAD2/3 (pSMAD2/3) is not detected in LECs during the first 24 hours after lens injury *in vivo*, and this readout of canonical TGFβ signaling is not robustly detected in remnant LECs until 72 hours PCS, likely due to the need of injured LECs to upregulate the genes needed for TGFβ activation (199). These likely include the matrix metalloproteases MMP2 and MMP9, whose expression upregulates following LEC injury, potentially leading to cleavage of the LAP protein to release active TGFβ (49, 173, 227, 228). In addition to MMPs, mechanical injury resulting from surgery can significantly increase the influx of blood proteins via opening of the BAB including proteases (49), such as cathepsins (229), plasmins (230), and ADAM17 (231), all of which can cleave latent TGFβ. Interrogation of the Lens Injury Response Time Series (LIRTS) viewer, which is a compendium of RNAseq analyses of mouse LECs following lens fiber cell removal surgery, also found that remnant LECs upregulate the transcripts encoding these proteases at 48H PLI, coinciding with the first detection of pSMAD2/3 in remnant LECs (208). Beyond protease activity, injured LECs upregulate the protein levels of αVβ8 integrin, and this is required for TGFβ activation PLI (209). Notably, fibrotic transformation of LECs can be blocked by treatment with functional blocking αVβ8-integrin antibodies, suggesting that this is a viable approach to the prevention of myofibroblast formation (and potentially fibrotic PCO pathogenesis) following cataract surgery (209).

Notably, this result creates a contradiction in the literature on which TGFβ isoforms are responsible for LEC EMT following lens injury *in vivo*. The groundbreaking work of the McAvoy laboratory first discovered that LEC EMT is likely controlled by TGFβ signaling via treatment of lens epithelial explants with purified active TGFβ1, TGFβ2 or TGFβ3, and suggested that TGFβ2 was the most potent isoform *in vitro* (232). Later, it was realized that TGFβ2 is the most abundant TGFβ isoform in the uninjured eye, where it is found at high levels in a latent form in the ocular humors (233). This led to the concept that fibrotic transformation of LECs is triggered by exposure of LECs to this high level of TGFβ2 upon breach of the lens capsule (89, 234) and exposure of this latent molecule to proteases such as MMPs (235). However, it is now apparent that ocular TGFβ2 in healthy eyes is likely to be at least in part produced by the lens as uninjured LECs express this gene at high levels endogenously, so naïve LECs are constantly exposed to latent TGFβ2 (208, 233, 236, 237). Further, αVβ8-integrin, which is required for TGFβ signaling in LECs following lens injury, is only capable of activating TGFβ1 and TGFβ3, as the LAP of TGFβ2 lacks the RGD sequence needed for integrin-mediated TGFβ activation (209, 238). Notably, injured LECs upregulate the expression of both TGFβ1 and TGFβ3 between 24 and 48 hours PL1, which corresponds to the first detection of pSMAD2/3 in remnant LECs at 48 hours PCS (183, 208, 209). Additional investigations into the function of the TGFβ1 and TGFβ3 produced by LECs in the fibrotic response of LECs to lens injury are thus needed.

Further complicating this picture, macrophages closely associate with the lens following cataract surgery/lens injury from 24 hours PLI until at least a week or more PLI *in vivo*, which likely derive from a mixture of resident ocular phagocytic cells (172, 239–241) and those arriving from the circulation (199). Initially, these macrophages appear to have a pro-inflammatory M1 phenotype which are best known for their ability to engulf tissue debris and bacteria, but later M2 macrophages (242), which in other systems produce active TGFβ to quell the inflammatory response are detected (243) although their role in inducing the fibrotic response of LECs is currently unclear as cellular ablation of circulating macrophages did not appear to ameliorate capsular bag fibrosis following lens injury in rats (244).

### Creation of fibrotic matrix

By 24 hours PLI, remnant LECs begin to upregulate the expression of numerous extracellular matrix (ECM) molecules typically associated with tissue fibrosis including fibronectin, type I collagen, ECM1, and tenascin C (183). Fibronectin deposition around injured LECs particularly appears to play a key role in myofibroblast production and persistence PLI (185). Deletion of the fibronectin gene from mouse LECs did not affect the initiation of TGFβ signaling at 48 hours PLI, but it blocked sustained TGFβ signaling at 72 hours PLI and beyond, greatly reduced injury-induced LEC proliferation, and the assembly of fibrotic ECM (**Figure 4**) (185). In other systems, fibronectin is established to play multiple roles in the fibrotic response including serving as a pioneering ECM needed to trigger other ECM molecules to assemble into matrix, and as a binding partner for numerous integrins where it mediates both cellular attachment and cellular signaling (245–248). Further, fibrotic ECM is known to contribute to the increased stiffness of fibrotic tissues, and this stiffness is essential for sustained TGFβ responses as stiff ECM induces mechanical strain and can help position latent TGFβ near MMPs, facilitating the liberation of active TGFβ (31, 249). This role of fibrotic ECM in the myofibroblast production/persistence needed for ASC and fibrotic PCO pathogenesis is supported by key roles for other fibrotic ECM components such as Tenascin C (TNC) (250), SPARC (251), lumican (252), and vitronectin (253). In murine lens injury models, TNC mRNA levels sharply increase by 24 hours post-injury in wild-type lenses (183), whereas TNC-null (KO) mice display markedly diminished EMT-like LEC elongation, reduced capsule wrinkling, and a delayed transition to fibroblast-like morphology, even at day 10 PLI (250). Moreover, in other tissues, TNC modulates YAP/TAZ signaling through integrin α5β1, influencing mechanotransduction and fibrotic responses (254), pathways that likely converge with TGFβ-SMAD signaling in PCO contexts. In addition, SPARC (Secreted Protein Acidic and Rich in Cysteine), a matricellular protein also involved in cell–ECM interactions, collagen deposition, and TGFβ signaling modulation, also plays a role in the lens injury response (251, 255, 256). SPARC-null mice develop early-onset lens opacification and exhibit aberrant ECM organization, characterized by reduced laminin and fibronectin deposition (257). *In vitro* studies using LECs from SPARC-deficient animals reveal exaggerated responses to exogenous TGFβ, including heightened expression of fibronectin and αSMA, suggesting that SPARC serves as a negative regulator of TGFβ-driven EMT in the lens (49, 257). Moreover, glucocorticoid stimulation via dexamethasone upregulates SPARC while concomitantly suppressing fibronectin and collagen IV, pointing toward a protective, homeostatic role in restraining fibrosis (49, 258).

Another critical component is lumican, a proteoglycan identified in post-mortem PCO specimens, which regulates TGFβ bioavailability (252). Lumican-null mice demonstrate delayed αSMA expression post-injury due to defective LTBP-1 sequestration in the ECM (252). Finally, vitronectin synergizes with fibronectin to promote fibroblastic phenotypes *in vitro* in cornea (259) and lens (253). LECs cultured on vitronectin substrates exhibit elongated morphologies, elevated αSMA, and nuclear Smad2/3 localization, phenotypes absent when cultured on laminin, where LECs maintain a more typical epithelial phenotype, suggesting that vitronectin not only supports cellular adhesion and migration but also provides biochemical cues that promote EMT and fibrotic transformation (253). The ED-A domain of fibronectin and vitronectin-integrin interactions collectively enhance TGFβ responsiveness and reinforce a pro-fibrotic microenvironment (260).

### The role of other pathways in the onset of fibrotic response in PCO

In addition to canonical TGFβ signaling and fibrotic matrix-mediated events, several other signaling cascades play critical roles in orchestrating the fibrotic response in residual LECs following cataract surgery (261). In other systems, canonical Wnt signaling synergizes with TGFβ signaling to enhance fibrotic responses (262, 263), and this pathway is first activated in LECs at 12 hours PLI and is found in most if not all fibrotic capsule associated cells (CACs) at 5 and 9 days PLI (261). Recently, several reports suggest that this Wnt signaling plays a crucial role in the LEC injury response (198, 261, 264, 265), which expands the gamut of pathways that could be manipulated to block the production of myofibroblasts needed for PCO pathogenesis.

Moreover, platelet-derived growth factor (PDGF) signaling is also upregulated in injured LECs likely due to the significant upregulation of the expression of the PDGF ligands, PDGF-A and PDGF-B, and their receptors (PDGFRα and PDGFRβ) (266, 267). PDGF signaling primarily promotes cell proliferation and migration via downstream PI3K/AKT and MAPK/ERK pathways (268). Although not traditionally considered a central fibrotic driver, emerging evidence indicates that PDGF signaling contributes to the production of fibrotic proteins and enhances TGFβ–mediated EMT in a supportive role (269–271).

Furthermore, LECs require BMP signaling for their initial development and long-term cellular phenotype (272, 273). In other cell types, BMPs can act as antifibrotic agents by opposing TGFβ–driven EMT and fibrosis (274–276). In injured LECs, BMP signaling is frequently suppressed as these cells convert to a myofibroblast phenotype, perhaps through the upregulation of Gremlin 1, an endogenous BMP antagonist (277, 278). In adults, Gremlin1 mRNA levels transiently upregulate over 300-fold in CACs at 48 hours PLI, while Gremlin1 protein is detected in CACs from 24 hours through 5 days PLI (185). Gremlin1 protein upregulation is attenuated in fibronectin conditional knockout lenses, which fail to either deposit fibrotic ECM or sustain canonical TGFβ signaling PLI, while the injury-induced fibrotic response (including TGFβ signaling) is restored in fibronectin null lenses treated with exogeneous Gremlin 1 (185). Another study found that Gremlin1 knockdown ameliorated PCO in rats, likely via effects on ERK, AKT, BMP and TGFβ signaling (279). While these data can suggest Gremlin1 functions in parallel with TGFβ in LEC EMT responses, studies also found that exogeneous Gremlin1 did not rescue the PLI fibrotic response of lenses lacking αVβ8-integrin (209). Further, while itga5cKO lenses have a muted fibrotic response associated with reduced TGFβ signaling, Gremlin1 mRNA levels are modestly elevated in injured itga5cKO LECs at 48 hours PLI, suggesting compensation (280). As αVβ8 integrin’s primary role in the LEC fibrotic response appears to be TGFβ activation (209), this suggests that the burst of Gremlin1 expression in LECs PLI is needed to prime the TGFβ pathway PLI. However, further investigations are needed to place Gremlin1 into the gene regulatory network that establishes the fibrotic response of LECs PLI and answer whether Gremlin1 is directly involved in myofibroblast production PLI.

### Likely molecular mechanisms that produce the aberrant lens fiber cells associated with Soemmering’s ring and “pearl-like” PCO following cataract surgery

In the intact adult lens, central LECs are quiescent, while equatorial LECs remain in the cell cycle, slowly producing new fiber cells throughout life (281). While the early responses of LECs to lens injury appear dominated by the initial injury response and production of myofibroblasts (31), it is clear that not all LECs convert to myofibroblasts following injury, and in cases where the geometry of the lens capsule and lens cells is optimized following extracapsular lens extraction, regeneration of a transparent fiber cell mass post-surgery is possible (282). This approach was even used in a clinical trial of children undergoing surgery for congenital cataract (283), although most of these patients later underwent a second surgery as the regenerated lenses did not retain transparency long term (284). However, in aggregate, this suggests that residual LECs are the likely source of Pearl-like PCO and Soemmering’s ring cells that express numerous lens fiber cell markers, and their lack of transparency derives from their cellular disorganization driven by a lack of spatial cues needed for precise fiber cell organization (85, 186, 285). While relatively little direct work has been done on the mechanisms driving LEC differentiation to a lens fiber cell phenotype following lens injury, extensive research on the embryonic lens has revealed that this process is controlled by numerous growth factor cascades, with FGF signaling playing a dominant role in the phenotypic conversion of LECs to lens fiber cells (192, 285–288). As the FGF gradient responsible for fiber cell differentiation is likely retained in the eye PLI, and the posterior lens capsule is a known FGF depot (289–291), the pathways responsible for Elschnig’s Pearl and/or Soemmering’s ring formation after cataract surgery are likely to be similar to those driving lens fiber cell differentiation in normal lenses.

## Why does PCO still develop at appreciable rates years after surgery

PCO development during the first year post-surgery likely results from the LEC response to the numerous cell signaling pathways acutely induced by surgery leading to changes in cell behavior that induce them to enter the optical axis (30, 31, 199). However, PCO rates within the first year of surgery have greatly diminished over the past 10 years due to the widespread introduction of “square edge” hydrophobic IOLs that sequester remnant LECs and their derivatives in Soemmering’s ring which resides outside of the optical axis (73, 74, 107, 292). In spite of this, significant numbers of adult patients still develop clinically significant PCO in the first 5–10 years PCS (107).

As this late onset PCO is occurring many months or years after the pathways acutely activated by surgery have receded, this implies that even at extended times PCS, the cell populations initially produced during the LEC’s acute response to cataract surgery but sequestered within Soemmering’s ring are capable of escaping onto the posterior capsule at extended post-surgical times as other cellular sources are unlikely. This idea is supported by images that show PCO material emanating from breaches in the barrier set up by interactions between the optic and lens capsule either at the point where the haptic is attached to the optic (**Figure 3B**) or other locations around the optic (**Figure 3C**) (84).

Grossly, Soemmering’s ring is typically viewed as an opaque ring of cells sequestered outside of the visual axis (**Figure 3A**) by the IOL, where it does not interfere with vision (84). Morphological investigations suggest that it is largely comprised of disorganized lens fiber cells formed from residual LECs that are not removed by cataract surgery, with a smaller set of LECs that likely are driving the slow expansion of Soemmering’s ring at extended times PCS (37, 198, 293). Further, myofibroblasts are detected using cellular markers within Soemmering’s ring and at focal areas of the lens capsule anterior to the IOL consistent with fibrotic matrix deposition commonly associated with Soemmering’s ring material and regions near the capsulorhexis (198, 209, 293, 294).

Recently, our understanding of the molecular composition of Soemmering’s ring was enhanced by bulk RNA-Seq profiling of this structure from cadaver eyes obtained from 17 donors and the results compared to age-matched lens epithelial cells obtained from normal donors (198). This revealed that Soemmering’s ring cells still exhibit enhanced expression of numerous markers of immune responses including several proinflammatory cytokines, as well as genes consistent with elevated TGFβ and canonical Wnt signaling years following cataract surgery consistent with the sustained presence of myofibroblasts (and potentially immune cells) at extended times PCS (295). The differentially expressed genes are also enriched in markers of cell migration and cellular senescence, while this analysis also found sustained expression of lens epithelial markers consistent with the observation of cells within Soemmering’s ring, which exhibit LEC morphology at extended periods PCS (37, 198, 293).

Overall, the pathogenesis of late-onset PCO needs additional investigation as the physical reasons that cause the seal between the IOL and lens capsule to be compromised, allowing Soemmering’s ring cells to reach the posterior capsule, are largely unexplored. However, knowledge of the cell types found in Soemmering’s ring lead to potential cellular mechanisms that could drive the development of PCO years after cataract surgery (**Figure 2**).

### Role of LEC-derived Elschnig pearls on the posterior capsule

While it is recognized that Soemmering’s ring largely consists of aberrant lens fiber cells (37), these cells are unlikely sources of PCO as they are unable to proliferate and lose their organelles (including cell nuclei) as part of their terminal differentiation (296–298). Their migratory ability is also likely limited as fiber cells formed in the intact lens are only able to move their basal and apical tips slowly along the capsule/apical side of the lens epithelium respectively until they reach their counterpart from another lens quadrant to form the lens suture (299–301). However, it is common to detect a lens epithelial cell population adjacent to the lens capsule in Soemmering’s rings at extended times PCS, and these cells apparently retain their ability to divide and produce additional fiber-like cells as Soemmering’s ring is recognized to enlarge at later times PCS (37, 186, 198). Similar to what is described above for “early onset PCO”, these cells are expected to be subject to the same growth factor gradients within the eye as the intact lens, so LECs closer to the anterior capsule would be more likely to retain their LEC phenotype, while those more exposed to the high concentrations of FGF ligands found in the posterior chamber would be induced to form fiber-like cells (192, 285, 288). If these cells remain sequestered at the capsular bag periphery, this could contribute to Soemmering’s ring expansion, but that would be expected to occur only slowly due to a “contact inhibition” directed brake on cell proliferation (83). However, if the LECs escape their confinement and migrate onto the posterior capsule, they would first migrate and proliferate as they are relieved from contact inhibition (83, 302), then would be expected to induce lens fiber cell differentiation which would result in Elschnig’s pearls as the anatomical signals (and space) for normal fiber cell morphogenesis are absent (96, 303). This is likely to be a common cause of late-onset PCO as Elschnig’s pearls are highly light scattering (96, 304), and histological investigations of PCO often find morphological evidence that PCO material is “lens fiber-like” (90, 293, 303, 305). However, additional investigations using molecular markers and gene expression profiling are needed to establish the cellular identity of these cells definitively.

### Role of myofibroblasts that escape the Soemmering’s ring or capsulorhexis rim

Myofibroblasts, likely derived from LEC EMT, are rapidly produced in response to cataract surgery (7, 31, 153). There is significant evidence that at least a portion of these myofibroblasts persist in Soemmering’s ring and at the anterior capsule rim for years PCS using morphological and cell marker analyses (**Figure 3A**) (36, 142, 209, 293, 306). Notably, myofibroblasts have also been successfully isolated from Soemmering’s rings obtained from human cadaver eyes which underwent cataract surgery two decades prior and were confirmed to be capable of cell proliferation following dissociation from the IOL-capsule complex (36). In primary culture, these myofibroblasts were also capable of migration, matrix contraction and ECM remodeling (49, 307), showing their potential to contribute to fibrotic PCO if released from their sequestration at the capsular bag periphery. We have found that lens derived myofibroblasts are capable of producing their own TGFβ ligands as well as the proteins needed to activate newly synthesized latent TGFβ during their initial production in the mouse model of cataract surgery, and the production of fibrotic ECM by these cells would further reinforce the cell signaling needed for them to retain their myofibroblast phenotype (183, 209). In the human capsular bag model, lens cells can survive in serum-free medium for over 1 year and can effectively colonize the entire surface of the once cell-free posterior capsule (178). In addition, human capsular bag cultures maintained in serum-free medium after cataract surgery secrete various cytokines and growth factors such as interleukins, FGF, and VEGF (191, 308). Many of these molecules are involved in promoting cell proliferation, migration, and transdifferentiation of LECs to myofibroblasts (49, 308).

Overall, this evidence suggests that any breach of the seal between the square edge of the IOL and the lens capsule could release long sequestered myofibroblasts from their contact inhibition, where they could migrate onto the posterior capsule, increase in number, and produce fibrotic ECM while triggering capsular wrinkling which would result in the light scatter that would clinically manifest as late onset fibrotic type PCO (**Figure 2**).

### Role of remnant LECs trapped in Soemmering’s ring

While it is established that Soemmering’s ring often includes a layer of cells with morphological and molecular features consistent with a LEC phenotype, evidence is emerging that these cells often chronically express molecular markers consistent with cellular stress, including those known to be induced acutely in LECs upon injury (198). These include the upregulation of heat shock proteins (309), other oxidative stress response genes such as heme oxygenase 1 (310, 311), and depletion of intracellular antioxidants such as glutathione (312). Notably, oxidative stress is a potent inducer of EMT in LECs, acting through activation of ERK1/2 and Wnt/β-catenin signaling pathways, which drives loss of epithelial identity (e.g., Pax6 downregulation) and upregulation of fibrotic genes such as αSMA, collagen I, and fibronectin (313). Additionally, antioxidant supplementation such as with glutathione or catalase has been shown to suppress TGFβ-induced αSMA expression and fibrotic remodeling in lens explants (312). In addition to oxidative stress, advanced glycation end products (AGEs) accumulate with age in the lens capsule and are significantly more abundant in diabetic individuals (314, 315). AGEs exacerbate LEC stress and fibrosis by enhancing TGFβ2–driven EMT and activating receptor for AGE (RAGE)–dependent signaling, leading to increased αSMA and fibronectin expression (314, 316). Furthermore, AGE-modified lens capsules promote senescence and secretion of pro-fibrotic senescence-associated secretory phenotype (SASP) factors in cultured LECs, amplifying myofibroblast conversion in neighboring cells (317). These findings suggest that LECs within Soemmerring’s ring exist in a sensitized, pro-stress state due to chronic oxidative imbalance and injury signals, which may lower the barriers to migration and prime them toward matrix remodeling and myofibroblast transdifferentiation over extended postoperative periods. While the cellular constituents of Soemmering’s ring provide potential sources of late-onset PCO, their ability to persist and eventually migrate onto the posterior capsule may also be shaped by the surrounding ocular environment.

### Influence of the lens capsule on late onset PCO

The no space/no cells concept of PCO prevention postulates that close contact between the lens capsule and IOL is essential to sequester cells from the visual axis following cataract surgery (88). In the intact lens, the lens capsule continually thickens throughout life due to the secretion of its component proteins by the lens cells it encompasses (318, 319). Cataract surgery disrupts this homeostatic mechanism, with either no cells found in association with this basement membrane ECM (within the visual axis) or the attached cells are not fully normal (ie the components of Soemmering’s Ring) at the IOL/capsule interface (320, 321). As prolonged expression of matrix metalloproteases and other degrading enzymes are present in the eye following cataract surgery (322), while new basement membrane protein synthesis is disrupted, it would be expected that the lens capsule would change properties at extended times PCS, which could compromise IOL-lens capsule interactions, allowing the escape of cells onto the posterior capsule where they have the potential to contribute to light scatter and late onset PCO (134, 322).

Additional evidence from human capsular-bag experimental systems indicates that the lens capsule is not simply a passive substrate for cell adhesion but can act as a long-acting reservoir and modulator of growth-factor signaling, with important implications for late-onset PCO (323). In human capsular bag model, brief exposure to TGFβ (for only a few days) produced persistent fibrotic changes that continued for weeks after ligand removal, reflecting both *de novo* TGFβ production by LECs and the ability of the capsule to adsorb and re-present TGFβ, thereby prolonging profibrotic signaling (323). Likewise, FGF-2 is also bound to the capsule matrix as perlecan and other heparan sulfate proteoglycans (HSPGs) concentrate matrix-bound FGF-2 at the epithelial–capsule interface, establishing spatial gradients, and MMP-2–dependent release of FGF-2 from the capsule sustains LEC viability and signaling activity (324). Collectively, these data support the concept that transient postoperative growth-factor surges can be converted into long-term, capsule-mediated signaling that maintains or re-activates LEC proliferation/differentiation long after acute wound healing has subsided, contributing to the pathogenesis of late-onset PCO.

### Persistent inflammation as a driver of late-onset PCO

Cataract surgery both induces remnant LECs to acutely express pro-inflammatory cytokines (199, 325) and opens the blood-aqueous barrier to allow the influx of serum proteins and immune cells from the circulation (326). In most cases, this acute inflammation subsides days or weeks PCS as the wound healing responses recede (199, 325). However, numerous reports suggest that eyes subjected to cataract surgery still exhibit signs of low-level inflammation (325, 327) and a recent extensive study detected elevated inflammatory cytokine levels in aqueous humor collected from cadaver eyes that had undergone cataract surgery years earlier (295). Further, cells isolated from Soemmering’s ring years following cataract surgery overexpress numerous pro-inflammatory cytokines compared to naïve LECs consistent with pseudophakic eyes experiencing chronic low-level inflammation (198). In other fibrotic conditions, it is established that inflammation can trigger tissue fibrosis via a number of mechanisms including chronic production of active TGFβ by inflamed tissues (328, 329). Thus, low level chronic inflammation may synergize with other changes in the ocular environment including other ocular surgeries, transient uveitis, onset of age-related macular degeneration, or vitreous degeneration leading to release of stored TGFβ or other growth factors, diabetes etc. to “activate” quiescent LECs or myofibroblasts to seek weaknesses in the IOL-capsular barrier, leading to late onset PCO.

## Other long-term complications of cataract surgery are likely influenced by Soemmering’s ring

### Lens decentration and late capsular bag dislocation

Beyond long-term PCO, other complications following cataract surgery may also compromise visual outcomes. One such delayed, but serious, issue is the late dislocation of the IOL within the capsular bag, which occurs at an incidence between 0.1% to 9.1% depending on the patient population and time that the patient survives after their initial cataract surgery (330–332). This complication can present as decentration or tilting of the IOL within the capsular bag or subluxation of the capsular bag/IOL complex leading to displacement of the optic’s position within the visual axis resulting in vision compromising optical distortions (333–336). In more severe cases, the zonules holding the IOL- capsular bag complex in position within the eye completely rupture, leaving this structure to float freely within the eye which leads to a major disruption of the eye’s optical power and compromises the health and function of other ocular structures including the cornea, retina, ciliary body/trabecular meshwork, and iris if not surgically corrected (330, 333).

Late IOL dislocation appears to be caused by a variety of mechanisms. First, the zonules, acellular elastic fibrils that attach the lens to the ciliary muscles, stabilize the position of both the natural lens and the pseudophakic IOL/capsular bag complex within the eye (337, 338). These first form in late eye development likely via the assembly of matrix produced by the ciliary complex (and perhaps the lens) and its incorporation into the capsule via a largely unknown process (337). In adult eyes, both the ciliary margin and LECs continue to express the genes needed for elastic fiber synthesis but how this influences zonular integrity across the lifespan is unknown (339, 340). Aging is often associated with the gradual deterioration of zonular fibers, and this process may accelerate in eyes where the natural lens is disrupted by cataract surgery (338, 340). Progressive age-associated zonular weakness may be exacerbated by underlying conditions such as diabetes mellitus, retinitis pigmentosa, or pseudoexfoliation syndrome and other connective tissue disorders (341). Additionally, previous ocular trauma or surgeries, such as vitreoretinal procedures, also increase the risk of zonular instability (334, 342), possibly due to elevations in matrix-degrading enzymes such as MMPs (343) and surgically induced biomechanical stress (344). While the role of lens cells in sustaining zonular integrity into old age is currently unknown, it is tempting to speculate that the cellular composition of Soemmering’s ring and its ability to participate in any processes needed for zonular health would influence whether zonules fail at prolonged times following cataract surgery.

Another factor that likely contributes to the pathogenesis of late IOL dislocation is the number, position, and organization of myofibroblasts within Soemmering’s ring and associated with the anterior capsule (36). The smooth muscle-like phenotype of myofibroblasts allows them to contract the fibrotic matrix and basement membranes to which they attached leading to centripetal tractional forces which would put strain on the zonules leading to their rupture (36). Notably, the presence and cellular activity of myofibroblasts isolated from IOL-capsular bag complexes surgically removed from eyes which developed IOL dislocation over 20 years following cataract surgery supports this pathogenic mechanism (36).

### Dead bag syndrome

The lens capsule (“the bag”) is an acellular basement membrane whose component proteins are produced by both lens epithelial and fiber cells, although capsule production by LECs is more robust leading the anterior lens capsule to be thicker than the posterior lens capsule (87, 289). As lens capsule components turn over slowly (if at all) (345, 346), and capsule material is continuously secreted by lens cells across the lifespan, the lens capsule continues to thicken with age (318, 319, 347), potentially influencing the pathophysiology of presbyopia (348–350). Extracapsular lens extraction with IOL implantation relies on the exceptional stability of the lens capsule compared to other basement membranes (which typically fully turn over every few days or weeks) as the removal of the lens fiber mass and molecular changes in the LECs presumably disrupt the normal processes ensuring lens capsule health (320, 321).

However, there is evidence that the cells found in Soemmering’s ring are essential to maintain the lens capsule and IOL position at extended times PCS (37). “Dead bag syndrome” is an emerging clinical entity characterized by the presence of a diaphanous capsular bag with a clear periphery (ie, lacking Soemmering’s ring) even many years after surgery (87, 351, 352). Coincident with this, the capsular bag itself loses its normal tautness even when the zonules are intact, allowing the position of the IOL to shift within, or even fall out of, the bag (352), via a peripheral bag defect. Unlike early IOL dislocations, which typically result from inadequate IOL fixation/position at the time of surgery, dead bag syndrome is believed to result from late-onset degenerative changes in the capsular bag (352). Notably, these changes are not associated with significant capsular fibrosis or contraction but rather with a gradual deterioration in the biomechanical properties of the bag, as well as evidence of capsular delamination shown in histopathological assessment in suspected cases (87). Currently, the natural history of Dead bag syndrome is understudied due to its relative rarity, so it is not definitively known if it occurs from a failure to initially form a Soemmering’s ring, or its later degeneration. It is noteworthy that polishing of the inner surface of the capsule during cataract surgery, just before IOL implantation, in an attempt to prevent capsular bag fibrosis and opacification is not uncommon. However, removal of all LECs during this procedure is unlikely, as polishing of the anterior capsular rim and equator is usually not possible, as this region is not readily visible or accessible to the surgeon. However, the phenotype of Dead bag syndrome does suggest that Soemmering’s ring cells retain their ability to support the long-term properties of the equatorial lens capsule. Notably, RNAseq profiling of Soemmering’s ring cells years after cataract surgery did find enhanced expression of the protease inhibitors Timp1 and SerpinE1 compared to naïve LECs, which may prevent the action of capsule destroying proteases (198). Further, Soemmering’s ring cells continue to express key components of the lens capsule, including collagen IV subunits, for years PCS (198). Overall, the pathogenesis of Dead bag syndrome suggests that Soemmering’s ring contributes to the long-term success of cataract surgery and that PCO prevention methods that focus on the ablation of all residual LECs from the capsular bag at the time of surgery could have unintended negative consequences at extended times PCS.

## In conclusion

The cells responsible for early-onset PCO form in direct response to phenotypic/molecular changes that LECs (and perhaps immune cells) undergo in response to cataract surgery (31, 32). These cells then create clinically significant PCO when they migrate onto the posterior capsule due to both their intrinsic ability to scatter light and in fibrotic PCO, their ability to compromise lens capsule transparency via fibrotic ECM deposition and capsular wrinkling (49). In contrast, late-onset PCO likely forms when LEC-derived cells and/or immune cells, sequestered within Soemmering’s ring (or residing on the remnant anterior capsule) for months or years after acute wound healing recedes, escape onto the posterior capsule due to a combination of physical disruption of capsule/IOL optic adhesion and reactivation of cell migration/proliferation pathways. Further, other long term (10–20 years PCS), but currently rare, side effects of cataract surgery are now emerging including late capsular bag dislocations, IOL decentration, and Dead bag syndrome, are also likely influenced by the cellular composition of the Soemmering’s ring or its failure to form (36). In aggregate, future investigations into the cellular composition of Soemmering’s ring, factors that contribute to its formation and persistence, as well as the mechanisms that induce (or allow) its components to enter the optical axis at extended times PCS will reveal information valuable to ensure the long-term efficacy of cataract surgery.

## Future directions

Despite major advances in surgical techniques and IOL design that have significantly reduced the incidence of early-onset PCO, late-onset PCO continues to present a substantial clinical challenge. A critical unanswered question is what destabilizes the capsule–IOL barrier years after cataract surgery, allowing Soemmering’s ring cells to escape onto the posterior capsule. Even in cases where hydrophobic square-edge IOLs and ideal capsulorhexis geometry create robust early barriers, clinically significant PCO still arises after 5–10 years. This suggests that long-term biomechanical or biochemical remodeling may compromise capsule–IOL adhesion, whether through gradual weakening of the capsular bag, chronic low-level inflammation altering ECM composition, or remodeling of contact points at the haptic–optic junction. Understanding the processes that erode this barrier is pivotal, as it represents the gateway event that permits late PCO initiation.

In addition, our lab has generated extensive datasets capturing the temporal sequence of cellular and molecular changes following lens injury, as well as the consequences of removing key genes on these dynamic responses. Building on these resources, future work should focus on constructing gene regulatory networks that integrate both acute injury-induced pathways and long-term survival or remodeling programs. Such models will enable a more comprehensive understanding of how early wound-healing signals set the stage for chronic changes, and how gene-specific perturbations alter the trajectory of PCO pathogenesis. This systems-level approach will be crucial for bridging acute responses with late-onset complications, and for identifying intervention points to prevent long-term PCO.
